# FORTITUDE IN DIVERSITY: MINORITY ISSUES AND INTEGRATIVE APPROACHES TO COGNITIVE HEALTH IN OLDER ADULT POPULATIONS

**DOI:** 10.1093/geroni/igae098.0776

**Published:** 2024-12-31

**Authors:** Ladan Ghazi Saidi, Christina Miyawaki

**Affiliations:** Chair; Co-Chair

## Abstract

First, Dr. Ghazi Saidi, an associate professor at the University of Nebraska at Kearney and the symposium chair, will discuss cognitive health disparities and interventions aimed at improving the cognitive health of older adults in minority communities. She will also present the results of a scoping review on cognitive health, including demographic and lifestyle factors that impact cognitive health in older adults living in rural areas. Next, Jeein Jang, a PhD candidate at the University of Massachusetts Boston, will present a cross-sectional study exploring the associations between neighborhood characteristics and activity engagement, with a focus on different activity domains and race/ethnicity, using data from the 2018-2020 Health and Retirement Study. Michelle Xue, a PhD student at Duke University, will introduce a dementia prevention program specifically designed for older Chinese Americans at high risk for dementia, aimed at overcoming barriers such as language and transportation. Maki Karakida, another PhD candidate at UMass Boston, will present on the relationship between work/retirement status and sleep health, emphasizing the impact of race/ethnicity and nativity, particularly in African American and Hispanic populations, among 41,924 Americans aged 50+, showing that intersections of race/ethnicity and nativity significantly influence sleep quality. Lastly, Dr. Miyawaki, an associate professor at the University of Houston, will discuss a cognition program conducted in Houston by Vietnamese-American health professionals and university students, which addresses the gap in dementia care using the Cultural Exchange Model (CEM) to enhance dementia literacy.

